# Interaction between *N*^6^-methyladenosine (m^6^A) modification and noncoding RNAs in cancer

**DOI:** 10.1186/s12943-020-01207-4

**Published:** 2020-05-22

**Authors:** Yi Chen, Yu Lin, Yongqian Shu, Jing He, Wen Gao

**Affiliations:** Department of Oncology, The First Affiliated Hospital of Nanjing Medical University, 300 Guangzhou Road, Nanjing, 210029 China

**Keywords:** m^6^A modification, Noncoding RNAs, Cancer

## Abstract

As a critical internal RNA modification in higher eukaryotes, *N*^6^-methyladenosine (m^6^A) has become the hotspot of epigenetics research in recent years. Extensive studies on messenger RNAs have revealed that m^6^A affects RNA fate and cell functions in various bioprocesses, such as RNA splicing, export, translation, and stability, some of which seem to be directly or indirectly regulated by noncoding RNAs. Intriguingly, abundant noncoding RNAs such as microRNAs, long noncoding RNAs, circular RNAs, small nuclear RNAs, and ribosomal RNAs are also highly modified with m^6^A and require m^6^A modification for their biogenesis and functions. Here, we discuss the interaction between m^6^A modification and noncoding RNAs by focusing on the functional relevance of m^6^A in cancer progression, metastasis, drug resistance, and immune response. Furthermore, the investigation of m^6^A regulatory proteins and its inhibitors provides new opportunities for early diagnosis and effective treatment of cancer, especially in combination with immunotherapy.

## Background

*N*^6^-methyladenosine (m^6^A), first described in 1974 [1, 2], is a well-known internal modification of messenger RNAs (mRNAs) and noncoding RNAs (ncRNAs); it is widely conserved among eukaryotes ranging from yeast, plants, and flies to mammals and even occurs in viral RNAs with a nuclear phase [3, 4]. As the most abundant and important mRNA modification in mammals, m^6^A modification accounts for approximately 50% of the total methyl-labeled ribonucleosides [5] and 0.1–0.4% of all adenosines in total cellular RNAs with about 3–5 m^6^A sites per mRNA [6]. m^6^A is enriched in the consensus sequence RRACH (where R: A or G and H: A, C, or U) and highly occurs in 3′ untranslated regions (3′-UTRs), stop codons, and internal long exons [4, 7], thus showing an effect on mRNA metabolism, including splicing, export, translation, and decay [8]. Notably, approximately 67% of 3′ UTRs with m^6^A peaks also contain binding sites for ncRNAs such as microRNAs (miRNAs) [7], thus suggesting a possible mechanism by which m^6^A and ncRNAs co-regulate target mRNAs through cooperation or competition.

In addition to mRNAs, m^6^A has also been discovered in diverse ncRNAs such as miRNAs, long noncoding RNAs (lncRNAs), circular RNAs (circRNAs), ribosomal RNAs (rRNAs), small nuclear RNAs (snRNAs), and small nucleolar RNAs (snoRNAs) [9, 10], and has been found to be essential for their metabolism and functions [11–14]. Furthermore, certain m^6^A regulatory proteins responsible for abnormal m^6^A modifications on ncRNAs are also involved in cancer cell proliferation, invasion, and drug resistance, suggesting a potential association between cancer and m^6^A ncRNA modification, and thus, offering a new opportunity for cancer diagnosis and treatment [15–17].

Although still in its infancy, efforts have been made to investigate the role of m^6^A in various types of ncRNAs. In this review, we generalize the recent progress in this field by our understanding of the interaction between m^6^A and ncRNAs with a focus on introducing the underlying regulatory mechanisms and biological consequences of m^6^A-modified ncRNAs, as well as the effects of ncRNAs on m^6^A mRNA modification. Finally, current knowledge and future perspectives of m^6^A in cancer diagnosis and treatment are also discussed, especially its relevant roles in immune response and immunotherapy.

### m^6^A writers, erasers, and readers

**Fig. 1:**
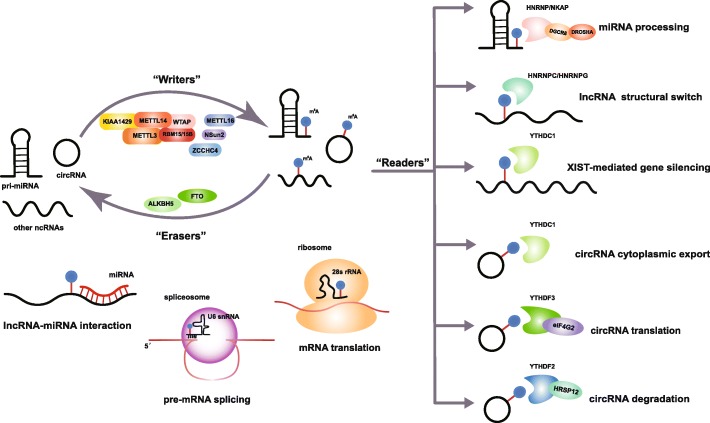
m^6^A modification on noncoding RNAs. m^6^A is deposited on ncRNAs by m^6^A writers consisting of the METTL3-METTL14-WTAP-RBM15/15B-KIAA1429 complex and many other methyltransferases such as METTL16, NSun2, and ZCCHC4. m^6^A can be removed by two m^6^A erasers: ALKBH5 and FTO. The function of m^6^A is mediated partly by m^6^A readers, which have been identified in members of the HNRNP family, the YTH domain-containing protein family, and NKAP. m^6^A is known to be involved in RNA biogenesis and functions, including miRNA processing, pre-mRNA splicing, RNA structure, RNA-RNA interaction, and RNA translation and degradation

The effect of m^**6**^A modification is determined by m^6^A regulatory proteins comprising m^6^A methyltransferases (m^6^A writers), m^6^A demethylases (m^6^A erasers), and m^6^A-binding proteins (m^6^A readers) (Fig. 1). m^6^A writers usually refer to the m^6^A methylase complex consisting of methyltransferase-like 3 (METTL3), methyltransferase-like 14 (METTL14), Wilm’s tumor-associated protein (WTAP), RNA-binding motif protein 15 (RBM15) and its paralog RBM15B, and KIAA1429 (also known as vir-like m^6^A methyltransferase associated [VIRMA]). As the catalytic core, METTL3’s methyltransferase domain is catalytically active [18], while METTL14 functions as an RNA-binding platform to enhance the methyltransferase activity by forming a heterodimer with METTL3 [18]. WTAP is considered as a key adaptor protein that stabilizes the METTL3-METTL14 complex [19], and RBM15/15B helps to recruit the complex to methylate specific sites through interaction with METTL3 in a WTAP-dependent manner [20]. KIAA1429 is an important part of the m^6^A methylase complex, but its molecular function remains elusive [21]. Although a multitude of m^6^A modifications are installed by the METTL3-METTL14-WTAP-RBM15/15B-KIAA1429 complex, other m^6^A methyltransferases such as METTL16 [22–24], NSun2 [25], and ZCCHC4 [26], which seem to be introduced independently, are also indispensable for m^6^A formation, especially in some ncRNAs.

As a dynamic and reversible RNA modification, m^6^A regulated by m^6^A writers and erasers exhibits the type of complexity seen with epigenetic modifications. Thus far, two demethylases have been identified as m^6^A erasers, namely alkB homolog 5 (ALKBH5) and fat mass and obesity-associated protein (FTO). However, in addition to m^6^A, FTO has also been shown to be responsible for the demethylation of *N*^6^, 2-O-dimethyladenosine (m^6^A_m_) in mRNAs and snRNAs [27, 28] and for the demethylation of *N*^1^-methyladenosine (m^1^A) in tRNAs [29] .

The m^6^A modifications that cause local changes in RNA structure can be recognized by selective RNA-binding proteins called m^6^A readers. The main m^6^A readers comprise the YT521-B homology (YTH) domain-containing protein family, including the nuclear YTHDC1 and the cytoplasmic YTHDC2, YTHDF1, YTHDF2, and YTHDF3. Other RNA-binding proteins, such as the heterogeneous nuclear ribonucleoprotein (HNRNP) family (HNRNPA2B1, HNRNPC, and HNRNPG) and NF-κB-associated protein (NKAP) can also affect RNA fate and cell functions through the specific recognition of m^6^A [30].

### Roles of m^6^A methylation on ncRNAs in cancer

#### MicroRNAs

miRNAs are a class of noncoding single-stranded RNAs with a length of 21–25 nucleotides; they regulate gene expression at the post-transcriptional level by forming the RNA-induced silencing complex (RISC), which binds to the 3′-UTR of target mRNAs, resulting in either translational inhibition or mRNA degradation [31]. In recent years, m^6^A has been observed in miRNAs that target oncogenes or tumor suppressors and appears to participate in cancer progression by affecting miRNA biogenesis or stability.

#### m^6^A **methylation** promotes miRNA processing

As elucidated by Alarcón et al., altered METTL3 could affect the steady state levels of not only mRNAs but also several miRNAs such as let-7e, miR-25, miR-93, miR-126, miR-221/222, and miR-4485. Further, they revealed that m^6^A methylation is an important mechanism in miRNA biogenesis [12]. In the nucleus, miRNAs are first transcribed as long primary miRNAs (pri-miRNAs), which are subsequently processed into precursor miRNAs (pre-miRNAs) by the microprocessor complex comprising the double-stranded RNA-binding protein DGCR8 and the RNase III endonuclease DROSHA, and then cleaved by Dicer into mature single-stranded miRNAs in the cytoplasm [32]. As shown in previous studies, DGCR8 initiates miRNA maturation by recognizing the junction between the stem of a pri-miRNA hairpin and the flanking single-stranded RNA, and then recruits DROSHA. DROSHA cuts the two strands of the stem at a position close to the base of the main stem loop to produce a pre-miRNA product [33, 34]. Intriguingly, this engagement and processing of pri-miRNAs is m^6^A-dependent. METTL3 marks pri-miRNAs with m^6^A modification, allowing DGCR8 to recognize and bind its specific substrates rather than other secondary structures present in transcripts, thereby promoting the maturation of miRNAs and leading to an increase in miRNAs in cells [12].

This finding provides a new molecular mechanism for METTL3 in tumor growth and metastasis. As elucidated by Han et al., METTL3 promotes the maturation of miR-221/222 in bladder cancer cells by interacting with DGCR8. This m^6^A-dependent miR-221/222 maturation results in PTEN reduction and ultimately leads to bladder cancer cell proliferation and tumor growth. Moreover, the association between high METTL3 expression and poor prognosis in bladder cancer implies that METTL3 may become a new prognostic factor and therapeutic target for bladder cancer [35]. In colorectal cancer (CRC), METTL3 promotes cancer cell migration and invasion by facilitating the biogenesis of miR-1246, which reverses the inhibition of the MAPK pathway through the downregulation of the tumor suppressor SPRED2, thus enhancing the metastatic capacity of CRC [36].

In addition to interaction with DGCR8 to facilitate pri-miRNA processing, METTL3 has also been proven to promote miRNA biogenesis by increasing the Dicer splicing of pre-miRNA. In non-small cell lung cancer (NSCLC), the upregulated level of miR-143-3p in brain metastasis tissue plays an essential role in cancer progression. It has been found to trigger the invasion of blood-brain barrier model and increase the angiogenesis of lung cancer by silencing vasohibin-1 (VASH1), which modulates VEGFA degradation and tubulin depolymerization. Further studies have shown that the cleavage of pre-miR-143-3p is m^6^A-dependent and this miR-143-3p/VASH1 axis can be positively regulated by METTL3. This finding shows the role of m^6^A in pre-RNA processing for the first time and provides a new target for patients with NSCLC with brain metastasis [37].

By facilitating the recognition and binding of DGCR8 to pri-miRNAs, METTL14 can also mediate miRNA maturation in an m^6^A-dependent manner and inhibit carcinogenesis and metastasis. In hepatocellular carcinoma (HCC), METTL14 has been proven to promote pri-miR-126 processing, while METTL14 depletion in HCC cells reduces m^6^A levels and miR-126 expression, leading to cancer cell migration and invasion [17]. In another study, METTL14 was found to suppress CRC progression by promoting the m^6^A-dependent maturation of miR-375, which could not only inhibit cancer cell proliferation by targeting Yes-associated protein 1 (YAP1), but also suppress cancer cell migration and invasion through the miR-375/SP1 pathway [38]. Furthermore, the downregulation of METTL14 is closely related to malignant progression and poor recurrence-free survival (RFS) and overall survival (OS) of patients, suggesting the potential role of METTL14 in predicting tumor metastasis and recurrence [17, 38].

Additionally, m^6^A readers are also involved in miRNA biogenesis. Alarcón et al. reported that HNRNPA2B1 could bind to m^6^A marks on pri-miRNAs and promote miRNA processing by recruiting DGCR8 [39]. Consistent with this finding, a recent study on NSCLC showed that the overexpressed oncogenic lncRNA LINC01234 regulated miR-106b-5p maturation by interacting with HNRNPA2B1 to promote pri-miR-106b processing, leading to cryptochrome 2 (CRY2) silencing and c-Myc expression. Intriguingly, activated c-Myc in turn bound LINC01234 promoter to increase its transcription, thus creating a positive-feedback loop that led to NSCLC cell proliferation and tumor growth [40].

Another m^6^A reader NKAP can also act as an adaptor for this m^6^A-dependent pri-miRNA maturation. In pancreatic duct epithelial cells, DNA hypomethylation of METTL3 promoter induced by cigarette smoke promotes METTL3 expression and thereby facilitates oncogenic pri-miR-25 processing in an NKAP-dependent manner. Consequently, mature miR-25/miR-25-3p activates the oncogenic AKT-p70S6K signal by inhibiting PH domain leucine-rich repeat protein phosphatase 2 (PHLPP2), thereby triggering the malignant phenotype of pancreatic cancer cells [41].

In glioblastoma metastasis, the m^6^A reader HNRNPC directly bound to pri-miR-21 to promote miR-21 expression, whereas silencing of HNRNPC downregulated miR-21 and suppressed the AKT-p70S6K pathway, leading to the expression of PDCD4 and thus inhibiting cell migration and invasion [42].

#### m^6^A **methylation** inhibits miRNA processing

Intriguingly, with no changes observed in the primary transcripts, several mature miRNAs were downregulated after m^6^A demethylase FTO knockdown [43], suggesting the negative influence of m^6^A on miRNA biogenesis and/or stability.

Consistent with this finding, the methyltransferase NOP2/Sun domain family, member 2 (NSun2) abolishes the gene silencing function of miR-125b by inhibiting the processing of pri-miR-125b2 into pre-miR-125b2 and the cleavage of pre-miR-125b2 into miR-125b [25]. Furthermore, this Nsun2-dependent miR-125b downregulation, which could be promoted by proteinase-activated receptor 2 (PAR2), may ultimately contribute to cancer cell migration and lymphatic metastasis in CRC due to the enhanced expression of the miR-125b target gene GRB2-associated binding protein 2 (Gab2) [44].

In LCC9 breast cancer cells resistant to endocrine, the upregulated HNRNPA2B1 seems to play a more complex role in miRNA transcriptome regulation. In detail, HNRNPA2B1 overexpressed in endocrine-resistant breast cancer cells reduces the sensitivity to 4-hydroxytamoxifen and fulvestrant by upregulating miR-1266-5p, miR-1268a, and miR-671-3p and downregulating miR-29a-3p, miR-29b-3p, and miR-222 [45]. Although previous studies have shown that HNRNPA2B1 can bind to pri-miRNAs and interact with DGCR8 for miRNA processing [39], the mechanism by which HNRNPA2B1 downregulates miRNA remains unclear.

Collectively, these findings indicate an important and complicated role of m^6^A in miRNA biogenesis, and the underlying mechanism of m^6^A-mediated miRNA downregulation remains elusive. Because m^6^A regulates mRNA decay, the inhibition of miRNA processing may result from the decreased expression of miRNA processing factors including DGCR8, DROSHA, and Dicer, although Berulava et al. found no significant change in their mRNA levels after FTO knockdown [43]. Another possibility is that m^6^A modifications on certain miRNAs may be selectively recognized by other reader proteins responsible for miRNA degradation or instability, an m^6^A-mediated mechanism that has been demonstrated in mRNAs and several ncRNAs [46]. In conclusion, these studies reveal that m^6^A is a critical regulator of miRNA levels, and the diverse interactions between miRNA and m^6^A regulatory proteins require further investigation.

#### Long noncoding RNAs

Transcripts of more than 200 nucleotides in length, but lacking functional coding capacity are defined as long noncoding RNAs (lncRNAs). In recent years, accumulating evidence demonstrates that lncRNAs control many aspects of gene expression and cellular biology at both transcriptional and post-transcriptional levels [47]. In an m^6^A mapping study, it was demonstrated that lncRNAs are also extensively m^6^A-modified [7].

#### m^6^A **methylation** acts as an RNA structural switch

RNA-binding proteins regulate the biological functions of their target RNAs by recognizing and binding to single-stranded RNA-binding motifs (RBMs), but these RBMs are often buried in local RNA structure, thus, avoiding interactions with RNA-binding proteins [48, 49]. Recently, m^6^A modification has been shown to act as a structural switch to affect RNA–protein interactions by modulating the structure of several RNAs, including metastasis-associated lung adenocarcinoma transcript (MALAT1) [13], an lncRNA whose dysregulation has been consistently associated with the progression of various cancers, and has been recognized as a biomarker in many studies [50]. Methylation of residues A2577 and A2515 in MALAT1, either encompassing or proximal to the RRACH consensus motifs, makes it easier for the surrounding RNA sequence to bind HNRNPC and HNRNPG, respectively. However, METTL3/METTL14 knockdown decreases the accessibility of MALAT1 to these proteins and thereby inhibits cell proliferation [13, 51]. Although the specific effects of HNRNPC/HNRNPG binding to MALAT1 remain unclear, the binding of HNRNPC/HNRNPG genomewide to transcripts has been shown to influence RNA expression and alternative splicing pattern [52–54]. In another study, METTL16 was identified as a triple-stranded RNA-binding protein of MALAT1 and was shown to affect cell proliferation in *Caenorhabditis elegans*, thus, suggesting a potential relationship between the METTL16–MALAT1 complex and the oncogenic activity of MALAT1 [55].

#### m^6^A **participates in** the lncRNA-mediated ceRNA model

Several authors have proposed lncRNA-mediated regulatory models, where lncRNA acts as a competitive endogenous RNA (ceRNA) to regulate the activity and biological function of miRNA [56–58]. In nasopharyngeal carcinoma (NPC), highly expressed m^6^A modifications increase the stability of the oncogenic lncRNA FAM225A, which promotes cancer cell proliferation, invasion, and migration by competitively absorbing miR-590-3p and miR-1275 like a sponge; thus, increasing their target integrin β3 (ITGB3) and activating the FAK/PI3K/Akt signaling pathway to promote NPC tumorigenesis and metastasis [59]. Similarly, the lncRNA LINC00958 promotes HCC lipogenesis and progression by sponging miR-3619-5p to increase hepatoma-derived growth factor (HDGF) expression, and this LINC00958/miR-3619-5p/HDGF axis could be positively regulated by METTL3, which increases the stability of LINC00958 in an m^6^A-dependent manner [60]. In NSCLC, METTL3-mediated m^6^A modification increases the levels and stability of MALAT1, which acts as a ceRNA to abolish the gene silencing function of miR-1914-3p, and thereby increases the downstream target YAP, leading to NSCLC metastasis and drug resistance to cisplatin [61]. These findings suggest the regulatory role of m^6^A in lncRNA-mediated ceRNA model related to cancer development and chemoresistance, and provides a new idea for cancer diagnosis and treatment.

In addition to enhancing the stability of lncRNA to ensure its function, m^6^A also appears to be directly involved in RNA-RNA interactions. In mammalian embryonic stem cells (ESCs), linc1281 regulates ESC differentiation by binding and sequestering let-7 family miRNAs associated with cellular pluripotency. Intriguingly, according to the recent research conducted by Yang et al., this lincRNA-miRNA interaction is m^6^A-dependent as m^6^A-deficient A-G mutant or METTL3 downregulation abolishes the direct binding of let-7 to linc1281 without changing the levels of linc1281 [62]. However, it remains unclear whether there are m^6^A reader proteins that affect the m^6^A-dependent lincRNA-miRNA interactions.

#### m^6^A promotes XIST-mediated gene silencing

During the development of female mammals, silencing of gene transcription on the X chromosome is mediated by the lncRNA X-inactive specific transcript (XIST), but the underlying mechanisms remain unclear [63]. WTAP, a partner of METTL3 that affects m^6^A deposition on targets [19, 64], has been identified as a XIST-associated protein in a proteomic analysis [65]. Another study showed that RBM15 and its paralogue RBM15B are also involved in m^6^A formation on XIST, which seem to interact with METTL3 in a WTAP-dependent manner to form the m^6^A methylase complex, and bind to the target sites using their RNA-binding domains [20]. Further functional screening showed that efficient deposition of WTAP and RBM15/15B is necessary for XIST-mediated transcriptional repression, while METTL3 knockdown impairs the XIST function [20, 66]; thus, supporting the promoting role of m^6^A in XIST-mediated gene silencing. Moreover, the reader protein YTHDC1 has been identified and shown to enable this m^6^A-dependent transcriptional repression, but the detailed mechanism remains unclear [20].

#### Circular RNAs

CircRNAs are the back-splicing products of precursor mRNA [67] and exhibit widespread and cell type-specific m^6^A methylation features. Despite sharing m^6^A writers (METTL3/ METTL14) and readers (YTHDF1/YTHDF2) that interact with mRNAs, many m^6^A sites on circRNAs are different from those on mRNAs. In detail, m^6^A-circRNAs are usually derived from exons in mRNAs with no m^6^A methylation, whereas mRNAs with m^6^A methylation on the same exons that constitute m^6^A-circRNAs show less stability when recognized by YTHDF2; this suggests that m^6^A may occur during or after circRNA formation, but the underlying mechanism needs further study [68].

#### m^6^A promotes cytoplasmic export of circRNAs

In CRC cells, m^6^A methylation has been shown to mediate the cytoplasmic export of a crucial oncogenic circRNA, circNSun2. As elucidated by Chen et al., m^6^A-modified circNSun2 in the nucleus could be recognized by YTHDC1 and exported to the cytoplasm, and circNSun2 then stabilizes HMGA2 mRNA through the formation of the circNSun2/IGF2BP2/HMGA2 complex, eventually leading to the invasion of CRC cells and liver metastasis. Furthermore, because m^6^A-modified circNSun2 has been found to be frequently upregulated in tumor tissues and serum samples of patients with CRC with liver metastasis, circNSun2 may become a new diagnostic/prognostic biomarker and a potential therapeutic target for CRCs with liver metastasis [69].

#### m^6^A **methylation** drives circRNA translation

Yang et al. revealed that m^6^A modifications on circRNAs can function as internal ribosomal entry sites (IRESs) for the cap-independent protein translation. This m^6^A-driven translation of circRNAs could be enhanced by METTL3 and METTL14, while FTO-mediated demethylation of m^6^A may reduce this translation process. Both the m^6^A reader protein YTHDF3 and the translation initiation factor eIF4G2 are required. Further analysis revealed that hundreds of endogenous circRNAs containing m^6^A modifications are translatable, suggesting the general function of circRNA, which expands the human transcriptome and provides a fresh view on m^6^A modification in protein translation [14].

#### m^6^A methylation mediates circRNA degradation

Studies have shown that m^6^A regulates mRNA degradation triggered by YTHDF2 mainly through two different pathways: deadenylation via the CCR4/NOT complex and endoribonucleolytic cleavage by the HRSP12-RNase P/MRP complex [46]. However, Park et al. reported that a subset of m^6^A-modified circRNAs bound by YTHDF2 was also selectively downregulated via the HRSP12-RNase P/MRP endoribonuclease complex [70], which revealed the regulatory role of m^6^A in circRNA degradation and suggested a possible molecular mechanism.

Regarding the function of circRNA in immunity, Chen et al. reported that endogenous m^6^A-modified circRNAs bound by YTHDF2 were incapable of stimulating RIG-I, which was probably due to YTHDF2-mediated degradation of circRNAs [71], whereas exogenous circRNAs lacking m^6^A modification activated the RIG-I pathway, leading to interferon production and induction of innate immunity [72]. This finding revealed the function of m^6^A in distinguishing endogenous circRNAs from exogenous circRNAs and suggested the potential role of YTHDF2-mediated circRNA degradation in inhibiting innate immunity. Further, the administration of unmodified circRNAs led to antigen-specific T cell activation, antibody production, and tumor inhibition in vivo [72], thus providing a new view for the role of m^6^A-modified circRNAs in antitumor immunity.

#### Small nuclear RNAs

The splicing of the precursor messenger RNA (pre-mRNA) is catalyzed by the multi-megadalton ribonucleoprotein (RNP) complex known as spliceosome, which comprises five small nuclear ribonucleoproteins (snRNPs) and numerous non-snRNP proteins [73]. These snRNPs consisting of small nuclear RNAs (U1, U2, U4, U5, and U6 snRNA) and proteins constitute the core components of various spliceosomal complexes [74]. Several post-transcriptional modifications have been identified on snRNAs as well as m^6^A modifications on U2, U4, and U6 spliceosomal snRNAs [75].

#### m^6^A **methylation** on U6 snRNA regulates pre-mRNA splicing

Spliceosomal U6 snRNA carries an m^6^A modification on position 43 [76]. Recently, studies have revealed that this m^6^A43 modification on spliceosomal U6 snRNA is mediated by the m^6^A RNA methyltransferase METTL16 and is highly possible to be introduced during early stages in U6 snRNP biogenesis [23, 24]. Interestingly, A43 is located in the evolutionarily conserved U6 sequence, which is base-paired to the 5′ splice site of the pre-mRNA in the first catalytic step of splicing [77]. Mutations of this modified position have been found to impede such snRNA-mRNA interactions, which are fatal in yeast [78], and the failed attempts to knockout METTL16 in mammalian cells [23] further suggest that the METTL16-mediated m^6^A43 modification of spliceosomal U6 snRNA may have critical roles in mRNA splicing and biogenesis.

#### Ribosomal RNAs

Ribosomes are necessary for mRNA translation in eukaryotes. Ribosomes are composed of four ribosomal RNAs known as 5S, 5.8S, 18S, and 25S (yeast)/28S (human) rRNAs, and approximately 80 ribosomal proteins arranged into a small subunit (SSU) and a large subunit (LSU) [79, 80]. The structure, assembly, and function of ribosomes may be affected by the chemical modifications on rRNAs [80]. Thus far, two m^6^A modifications have been identified on rRNA: one is m^6^A4220 modification on 28S rRNA, and the other is m^6^A1832 modification on 18S rRNA [75, 81].

#### m^6^A **methylation** on rRNA affects mRNA translation

Recently, a new human m^6^A methyltransferase, ZCCHC4, has been found to promote liver tumor growth by regulating ribosome translation through modulating m^6^A4220 on 28S rRNA. In patients with HCC, the expression of ZCCHC4 and m^6^A on 28S rRNA in cancer tissues was significantly increased as compared to that in the surrounding healthy tissues, whereas ZCCHC4 knockout in a xenograft mouse model eliminates m^6^A modification on 28S rRNA and then decreases global translation activity, which contributes to the inhibition of HCC cell proliferation and reduction in liver tumor size; thus, highlighting the essential role of m^6^A rRNA modification in mRNA translation and tumor progression [26]. Consistent with this finding, van Tran et al. confirmed ZCCHC4 as a 28S rRNA m^6^A4220 methyltransferase that acts only on rRNAs [82], and further studies on the structure and regulation of ZCCHC4 in m^6^A methylation of 28S rRNA showed that the specific binding and methylation of ZCCHC4 to substrates depended on the stem loop structure of 28S rRNA [83]. In addition, the findings that ZCCHC4 is located in nucleoli, where the ribosomes are assembled, and ZCCHC4 interacts with proteins involved in RNA metabolism provide a possible mechanism by which ZCCHC4 influences RNA translation by regulating ribosomal assembly and biogenesis [84].

METTL5 has also been confirmed as a novel m^6^A writer responsible for the m^6^A1832 modification of 18S rRNA [82], and METTL16 has also been shown to interact with 5.8S, 18S, and 28S rRNAs [55], which require further investigation in the future.

As described above, m^6^A modifications modulated by several m^6^A regulatory proteins produce multiple biological functions in ncRNAs (Table 1).

**Table 1:** Molecular mechanisms and biological functions of m^6^A in noncoding RNAs

Molecule	Mechanism	ncRNA	Biological function	Ref.
Writers				
METTL3	Promote pri-miRNA processing	miR-221/222	Promote cell proliferation in bladder cancer	[35]
	Promote pri-miRNA processing	miR-1246	Promote cell migration in CRC	[36]
	Promote pre-miRNA processing	miR-143-3p	Promote the brain metastasis of NSCLC	[37]
	Promote pri-miRNA processing	miR-25	Promote cell proliferation in pancreatic cancer	[41]
	Modulate the structure of lncRNA	MALAT1	Promote cell proliferation	[13]
	Stabilize lncRNA and enable the ceRNA model	LINC00958	Promote HCC lipogenesis and progression.	[60]
	Stabilize lncRNA and enable the ceRNA model	MALAT1	Promote NSCLC drug resistance and metastasis	[61]
	Promote lincRNA-let-7 interaction	Linc1281	Regulate pluripotency and differentiation of mESC	[62]
METTL14	Promote pri-miRNA processing	miR-126	Suppress HCC metastasis	[17]
	Promote pri-miRNA processing	miR-375	Suppress CRC progression	[38]
	Modulate the structure of lncRNA	MALAT1	Promote cell proliferation	[13]
WTAP, RBM15, RBM15B	Promote XIST -mediated gene repression	XIST	Gene silencing on the X chromosome	[65] [20]
NSun2	Suppress pri-miRNA processing	miR-125b	Promote cell migration in CRC	[44]
METTL16	Regulate pre-mRNA splicing	U6 snRNA		[24]
ZCCHC4	Promote mRNA translation	28S rRNA	Promote cell proliferation	[26]
Erasers				
FTO	Affect steady-state levels of several miRNAs	miRNAs		[43]
	Suppress cap-independent translation	circRNAs		[14]
Readers				
NKAP	Promote pri-miRNA processing	miR-25	Promote cell proliferation in pancreatic cancer	[41]
HNRNPA2B1	Promote pri-miRNA processing	miR-106b	Promote NSCLC cell proliferation and tumor growth	[40]
	Alter miRNAs transcriptome	miR-29a-3p, miR-29b-3p, miR-222, miR-1266-5p, miR-1268a, miR-671-3p	Promote endocrine resistance in breast cancer	[45]
HNRNPC	Promote pri-miRNA processing	miR-21	Promote cell migration and invasion in glioblastoma	[42]
HNRNPC, HNRNPG	Regulate RNA abundance and alternative splicing pattern	MALAT1	Promote cell proliferation	[13, 51]
YTHDC1	Promote XIST -mediated gene repression	XIST	Gene silencing on the X chromosome	[20]
	Promote cytoplasmic export of circRNAs	circNSun2	Promote cell invasion and liver metastasis in CRC	[69]
YTHDF2	Mediate circRNA degradation	circRNAs	Inhibit innate immunity	[68, 70–72]
YTHDF3	Drive cap-independent translation	circRNAs		[14]

### ncRNAs regulate m^6^A methylation on mRNAs in cancer

#### miRNAs and m^6^A formation on mRNAs

**Fig. 2:**
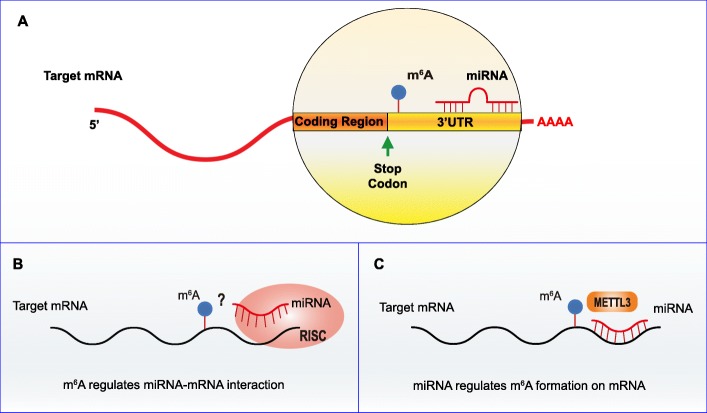
Association between m^6^A and miRNAs on 3′-UTRs of mRNAs. (A) m^6^A is more common on 3′-UTRs of mRNAs containing miRNA target sites. m^6^A is mostly deposited near the stop codon, whereas miRNA target sites are generally enriched in 3′ end of 3′-UTRs (B) m^6^A may regulate the binding and gene silencing function of the downstream-bound miRNAs. (C) miRNAs promote METTL3-mediated m^6^A formation on mRNAs through a sequence pairing mechanism

Previous studies have shown that 67% of 3′-UTRs of mRNAs with m^6^A peaks contain more than one miRNA binding site, and the overall distribution of these two groups in 3′-UTRs is inversely related. In 3′-UTRs containing these two groups, m^6^A peaks usually precede miRNA binding sites, thus indicating a possible interaction between m^6^A and downstream-bound miRNA [7] (Fig. 2). On the one hand, m^6^A on lincRNA promotes the binding of miRNA [62], allowing us to wonder whether m^6^A proximity to miRNA binding sites is involved in miRNA-mRNA interaction and thereby regulates miRNA-mediated transcript inhibition mechanism. On the other hand, the observation that higher expression of miRNAs is usually accompanied by a higher proportion of m^6^A-modified target transcripts suggests that miRNAs may regulate m^6^A abundance on their target transcripts [7]. Consistently, Chen et al. reported that miRNAs could modulate m^6^A formation on mRNAs by promoting the binding of METTL3 through a sequence pairing mechanism, which increased m^6^A levels and allowed the reprogramming of mouse embryonic fibroblasts (MEFs) into pluripotent stem cells [85].

#### lncRNAs regulate m^6^A modification on RNAs

**Fig. 3:**
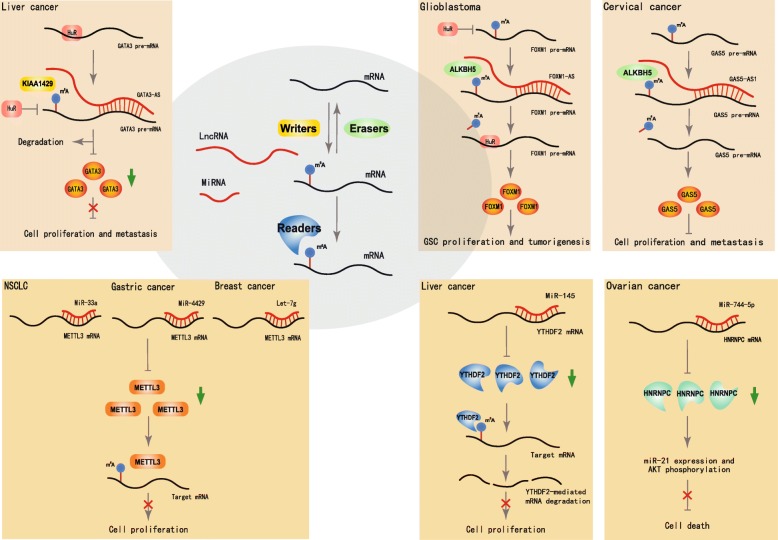
noncoding RNAs affect cancer progression and metastasis by regulating m^6^A modification of mRNAs

Recently, a class of antisense transcript-derived lncRNAs has been shown to play a role in cancer development by regulating the interaction between m^6^A regulatory proteins and their target mRNAs (Fig. 3). The writer protein KIAA1429 is highly expressed in HCC tissues and is closely correlated with poor prognosis in patients with HCC. KIAA1429 has been found to induce GATA3 mRNA degradation by modifying the 3′-UTR of GATA3 pre-mRNA with m^6^A, which abolishes the binding of HuR responsible for pre-mRNA processing and mRNA stability. Intriguingly, the lncRNA antisense to GATA3, GATA3-AS, could act as a guide to facilitate this KIAA1429-mediated m^6^A modification of GATA3 pre-mRNA, and thereby promotes the decrease in GATA3 expression, leading to the growth and metastasis of liver cancer [86].

In glioblastoma stem-like cells (GSCs), the m^6^A eraser ALKBH5 is highly expressed and predicts poor survival of patients with glioblastoma (GBM). As elucidated by Zhang et al., ALKBH5 promotes the tumorigenesis of GSCs by removing m^6^A from the FOXM1 nascent transcripts, which enhances mRNA stability and sustains FOXM1 expression. Additionally, the FOXM1-AS lncRNA, which is transcribed from the antisense strand of the FOXM1 gene, has been identified to promote this ALKBH5-FOXM1 interaction, and thereby, enhance FOXM1 expression and GSC proliferation [87]. Similarly, in cervical cancer (CC), lncRNA GAS5-AS1 is proven to interact with GAS5 and increase its stability by promoting the ALKBH5-dependent m^6^A demethylation, leading to the expression of the tumor suppressor GAS5, and thereby, inhibiting CC cell proliferation, migration, and invasion [88].

#### miRNAs regulate the expression of m^6^A regulatory proteins

miRNAs can also regulate m^6^A levels and biological functions by targeting the mRNAs of m^6^A regulatory proteins (Fig. 3). METTL3 is responsible for m^6^A modifications on mRNAs of several crucial oncoproteins related to cell proliferation, migration, and invasion in many cancers [89]. As elucidated by Du et al., miR-33a could prevent NSCLC progression by targeting the 3′-UTR of METTL3 mRNA and seems to be a promising target for treatment improvement for NSCLC [90]. Similarly, miR-4429, another potential therapeutic target in gastric cancer (GC), targets and downregulates METTL3 to inhibit m^6^A-induced stabilization of SEC62, which in turn inhibits cell proliferation and induces apoptosis in GC cells [91].

In breast cancer, miRNA let-7 g downregulates METTL3 expression by targeting its mRNA 3′-UTR, while hepatitis B X-interacting protein (HBXIP) improves METTL3 expression by inhibiting the function of let-7 g. Intriguingly, METTL3 could upregulate HBXIP in an m^6^A-dependent manner, which forms a positive feedback loop of HBXIP/let-7 g/METTL3/HBXIP, and thereby accelerates breast cancer cell proliferation [92].

In HCC, miR-145 inhibits tumorigenesis by targeting the mRNA of YTHDF2, resulting in suppressed YTHDF2 expression and decreased degradation of m^6^A-modified mRNAs and thus inhibiting HCC cell proliferation [93]. In addition, miR-744-5p leads to ovarian cancer cell death by targeting HNRNPC. The silencing of HNRNPC affects miR-21 expression and AKT phosphorylation, which ultimately contributes to apoptosis. It is worth mentioning that this effect is enhanced in combination with carboplatin treatment [94].

In summary, these findings revealed that ncRNAs can affect m^6^A modifications on mRNAs by targeting m^6^A writers, erasers, and readers in terms of both their expression and functions (Table 2), thus providing the foundation for further exploration of the roles of RNA epigenetic regulation patterns in cancers.

**Table 2:** Multiple functions of noncoding RNAs in m^6^A mRNA modification

ncRNAs	Mechanism	Molecule	Biological function	Ref.
miRNAs	Regulate m^6^A formation on mRNAs	METTL3	Promote cell reprogramming to pluripotency	[85]
GATA3-AS	Promote KIAA1429-GATA3 pre-mRNA interaction	KIAA1429	Promote cell proliferation and metastasis in HCC	[86]
FOXM1-AS	Promote ALKBH5-FOXM1 pre-mRNA interaction	ALKBH5	Promote GSC proliferation and tumorigenesis	[87]
GAS5-AS1	Increase GAS5 stability by interacting with ALKBH5	ALKBH5	Suppress cell proliferation and metastasis in cervical cancer	[88]
miR-33a	Inhibit METTL3 expression by targeting its mRNA	METTL3	Suppress cell proliferation in NSCLC	[90]
miR-4429	Inhibit METTL3 expression by targeting its mRNA	METTL3	Suppress cell proliferation in gastric cancer	[91]
Let-7 g	Inhibit METTL3 expression by targeting its mRNA	METTL3	Suppress cell proliferation in breast cancer	[92]
miR-145	Inhibit YTHDF2 expression by targeting its mRNA	YTHDF2	Suppress cell proliferation in HCC	[93]
miR-744-5p	Inhibit HNRNPC expression by targeting its mRNA	HNRNPC	Induce ovarian cancer cell death	[94]

### m^6^A as a diagnostic target of cancers

Given the critical roles of m^6^A in cancer cell proliferation, migration, invasion, and drug resistance, m^6^A modification and its regulatory proteins seem to be good diagnostic targets.

Circulating tumor cells (CTCs) derived from tumors can truly reflect the status and progression of the tumor. The finding that the levels of m^6^A are significantly elevated in CTCs from patients with lung cancer suggests that m^6^A in CTCs can be used as an early indicator to monitor and prevent cancer development and metastasis [16]. Furthermore, circRNAs showing a stable structure are promising biomarkers. In CRCs, the m^6^A-modified circNsun2, which has been proven to be positively associated with CRC cell aggressiveness, is frequently upregulated in serum and metastatic liver tissues, thus providing a novel diagnostic/prognostic predictor for colorectal liver metastasis [69]. Additionally, the early detection of m^6^A regulatory proteins may also be beneficial. In patients with bladder cancer, METTL3, which participates in cancer development by accelerating the m^6^A-dependent maturation of miR-221/222, is related to their poor prognosis [35]. In HCC, the methyltransferase METTL14 seems to be a good prognostic factor for cancer metastasis and recurrence because of its role in promoting the maturation of miR-126 that inhibits the metastasis of HCC [17]. Collectively, these findings provide new opportunities for the utilization of m^6^A in cancer diagnosis and prognosis.

### m^6^A and immunology of cancers

The maladjustment of m^6^A machinery provides interesting targets for tumor therapy, including emerging checkpoint blockade immunotherapy. FTO-mediated m^6^A demethylation has been found to regulate the occurrence and development of many cancers such as glioblastoma and breast cancer [95]. Recently, several selective inhibitors of FTO have been investigated, among which meclofenamic acid (MA) [96, 97] and MO-I-500 [98] were found to effectively suppress glioblastoma progression and breast cancer cell survival by inhibiting the catalytic activity of FTO. Notably, Yang et al. reported that FTO in melanoma cells not only promoted tumorigenesis but also mediated anti-PD-1 resistance. Depletion of FTO suppressed the expression of PD-1 (PDCD1), CXCR4, and SOX10 genes through m^6^A/YTHDF2-mediated mRNA decay and thus sensitized melanoma cells to interferon-gamma (IFN-γ) and anti-PD-1 blockade immunotherapy; this finding suggests that the combination of FTO inhibitors with anti-PD-1 treatment might be beneficial to reduce the resistance of melanoma to immunotherapy [99].

Recently, accumulating evidence has demonstrated the critical role of m^6^A in both innate and adaptive immune response [71], suggesting the potential effect of m^6^A on tumor immunology. T cells regulate the entire adaptive immune response, and the research conducted by Li et al. showed the regulatory role of m^6^A modification in naïve T-cell homeostasis and differentiation, indicating that m^6^A could be a potential target in antitumor immunotherapy and autoimmune disease therapy [100]. Regarding regulatory T cells (Tregs), a specialized T-cell lineage that is closely related to self-tolerance and immunosuppression, Tong et al. reported that m^6^A could maintain Treg functions by targeting the IL-2/STAT5/SOCS signaling pathway [101]. Because tumor-infiltrating Tregs restrict the tumor-killing functions of CD8+ T cells in tumor microenvironment, combining selective downregulation of m^6^A levels in Tregs with tumor immunotherapy may be an effective new therapeutic strategy.

Dendritic cells (DCs) are essential antigen-presenting cells that induce antigen-specific T-cell responses. Han et al. showed that m^6^A modification controlled DC function by enhancing the translation of lysosomal protease transcripts in a YTHDF1-dependent manner. Knockdown of YTHDF1 limited lysosomal proteolysis in DCs and enhanced the cross-presentation of tumor antigens, leading to better cross-priming of CD8+ T cells and therapeutic efficacy of PD-L1 checkpoint blockade; this finding suggests that YTHDF1 depletion combined with emerging checkpoint blockade or DC vaccination can serve as potential therapeutic targets for immunotherapy [102]. On the other hand, Wang et al. reported that METTL3-mediated m^6^A modification could facilitate the translation of CD40, CD80, and TLR4 signaling adaptor Tirap mRNAs in DCs by binding to YTHDF1, which not only promoted antigen presentation and DC-based T-cell responses but also stimulated cytokine production through the TLR4/NF-κB signaling pathway [103]. Consistently, another study on LPS-induced inflammatory response showed that METTL3 was positively correlated with the expression of inflammatory cytokines such as IL-6, IL-8, GRO, Gro-α, and RANTES, whereas METTL3 inhibition suppressed NF-κB and MAPK signaling pathways by affecting the alternative splicing of MyD88 [104] or the nuclear export of Traf6 mRNA [105]. Collectively, these findings revealed the flexible role of m^6^A in DC function and innate immunity, suggesting that the underlying mechanism of m^6^A in tumor immunology might be more complex and therefore needs further research.

## Conclusions

The dynamic and reversible m^6^A methylation has been shown to regulate post-transcriptional gene expression through several mechanisms. In addition to mRNAs, multiple types of ncRNAs also require m^6^A methylation for proper biogenesis, and utilize base modifications to tune structure and functions.

The effects of m^6^A modification are determined by m^6^A readers, writers, and erasers. New approaches and techniques are needed to investigate the distribution and functions of m^6^A modification in ncRNAs, as well as the associated m^6^A regulatory proteins in more detail, which could not only help us to fully understand the significance of m^6^A modification and their contribution to the highly dynamic cellular processes, but also to expand our scope of knowledge in the multilayered gene expression control mechanisms.

Dysregulation of m^6^A and its regulatory proteins is related to the diagnosis and prognosis of various diseases, including cancers, and these associated m^6^A regulatory proteins might represent interesting therapeutic targets. It is worth noting that the development of clinically applicable selective and effective inhibitors of FTO and other m^6^A regulatory proteins may provide new and more effective treatment strategies to treat cancer, especially when used in combination with emerging immunotherapies.

## Data Availability

All data generated or analyzed during this study are included in this published article [and its supplementary information files].

## Abbreviations

m^6^A: *N*^6^-methyladenosine
mRNAs: Messenger RNAs
ncRNAs: Noncoding RNAs
FTO: Fat mass and obesity-associated protein
3′-UTRs: 3′ untranslated regions
miRNAs: MicroRNAs
lncRNAs: Long noncoding RNAs
circRNAs: Circular RNAs
rRNAs: Ribosomal RNAs
snRNAs: Small nuclear RNAs
snoRNAs: Small nucleolar RNAs
METTL3: Methyltransferase-like 3
METTL14: Methyltransferase-like 14
WTAP: Wilm’s tumor-associated protein
RBM15: RNA-binding motif protein 15
ALKBH5: AlkB homolog 5
HNRNP: Heterogeneous nuclear ribonucleoprotein
NKAP: NF-κB-associated protein
RISC: RNA-induced silencing complex
pri-miRNAs: Primary miRNAs
pre-miRNAs: Precursor miRNAs
NSCLC: Non-small cell lung cancer
HCC: Hepatocellular carcinoma
MALAT1: Metastasis-associated lung adenocarcinoma transcript
ceRNA: Competitive endogenous RNA
